# Editorial: Increasing patient’s safe in colorectal surgery *via* real-time bowel perfusion using near infrared ICG fluorescence studies

**DOI:** 10.3389/fsurg.2022.922090

**Published:** 2022-09-14

**Authors:** Peter C. Ambe

**Affiliations:** ^1^ Chair of Surgery II, Witten/Herdecke University, Witten, Bergische Gladbach, Germany; ^2^ Department of General, Visceral and Colorectal Surgery, GFO Kliniken Rhein Berg, Witten, Bergische Gladbach, Germany

**Keywords:** ICG = near-infrared indocyanine green, anastomotic leak in colorectal surgery, patients safety, colorectal surgery, left colectomy

**Editorial on the Research Topic** Increasing patient’s safe in colorectal surgery *via* real-time bowel perfusion using near infrared ICG fluorescence studies by Ambe PC. (2022) Front. Surg. 9: 922090. doi: 10.3389/fsurg.2022.922090

Anastomotic leakage (AL) is the most feared complication in colorectal surgery and preventing this serious morbidity is a primary goal. Although the etiology of AL is multifactorial, three categories of risk factors can be identified. The first group includes “patient-related factors” like advanced age, male gender, obesity, concomitant diseases, etc. The second group is directly associated with the underlying pathology e.g., low rectal cancer and prior radiation, while the third group is surgery-related. The third group may include all perioperative aspects from preoperative preparation, surgical technique, postoperative management, etc and is therefore not limited to the expertise of the operating surgeon alone.

Anastomotic leakage is a complication that has probably been encouraged by almost every colorectal or gastrointestinal surgeon. Ever wondered why an AL develops even after creating a vital, tension-free and air-tight anastomosis? Maybe the perfusion was not as good as you conceived! Our judgement of bowel perfusion at the anastomotic site may not be always objective.

Over the last years objective real-time studies of bowel perfusion during the creation of an anastomosis has been increasingly reported. Fluorescent studies using indocyanine green (ICG) is one method of judging bowel perfusion during surgery. While the application of ICG is not new in medicine and surgery, its application in colorectal surgery is being advocated as a new standard with regard to evaluation of bowel perfusion.

In a recently published retrospective study Neddermeyer et al. (1) compared the outcomes of two cohorts with and without ICG imaging prior to colorectal or coloanal anastomosis following left-sided colectomy or rectal resection with respect to the rate of AL. Patients with benign (mostly diverticular disease) and malignant pathologies (colorectal cancer) were included in this study. The primary endpoint was the rate of AL.

The transection line was chosen by the leading surgeon based on bowel coloration in white-light, pulsation of end vessels and peristaltic waves prior to bowel transection. Then ICG was injected for perfusion studies. Per institutional protocol 5 ml ICG (5 mg/ml) was injected to judge the perfusion of the proximal colon after the anvil has been implanted. The degree of fluorescence of the colon was compared and judged with that of the small bowel. Using this algorithm, the future anastomosis site at the proximal colon was deemed as poorly perfused and was corrected in 12.9% of cases following ICG- imaging. Overall, a statistically significant lower rate of AL was documented in the ICG group compared to the non-ICG cohort (1.4 vs. 14.5%).

This study, albeit its retrospective design aimed at investigating the rate of AL in cases with ICG vs. no-ICG and represents one of the first studies of its kind. The main result confirms the importance of ICG imaging as a simple and effective means of reducing the risk of AL in colorectal surgery. This finding is even more compelling considering the fact that a correction of the transection line was performed in 12.9% in the ICG group. If perfusion was the sole reason for AL in colorectal surgery, which is not, this finding would have meant 12.9% more cases of AL in the ICG cohort.

**Figure 1:**
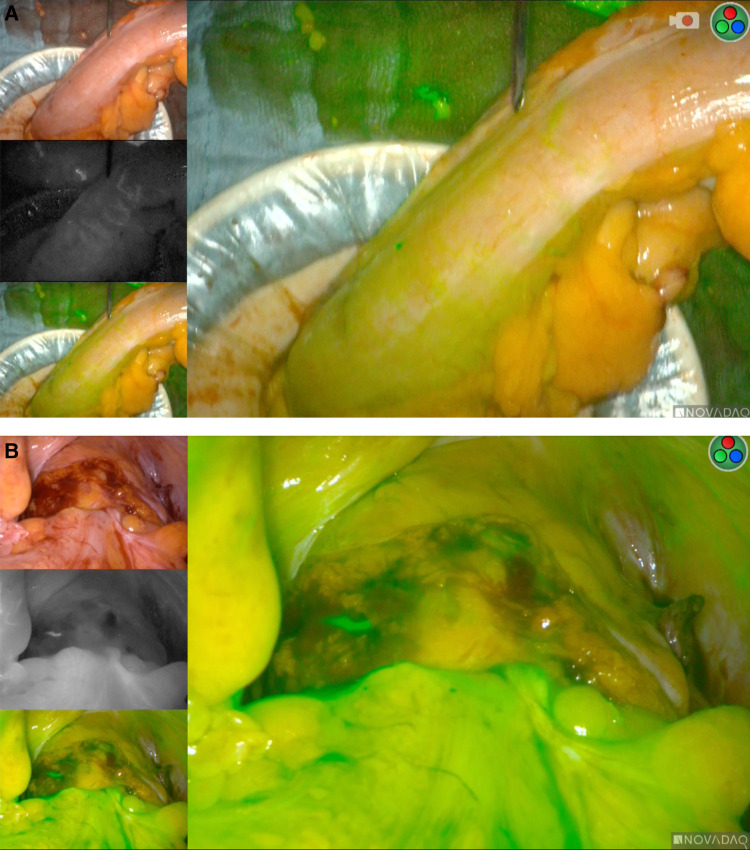
(**A**) real-time ICG fluorescence studies prior to bowel transection. (**B**) ICG-Studies during colorectal Anastomosis.

Despite the magnitude of the main finding from this study, some methodological aspects in the study by Neddermeyer et al. (1) may are worth discussing. My personal approach is to perform ICG prior to bowel transection (Figure 1A). This eliminates the need for correcting the transection line prior to anastomosis. Also, the perfusion of the distal bowel (usually the rectum) was not accessed by Neddermeyer et al. (1). The rectal stump could as well be poorly perfused, especially following radical resection for cancer. I therefore advocate a second perfusion study, again with 2.5 ml ICG just before complete closure of the stapling device (Figures 1A,B).

Despite different application methods, studying bowel perfusion objectively prior to creating an anastomosis and ensuring viable bowel perfusion is crucial in reducing the risk of AL. However, it must be clearly stated that, if sub-optimal perfusion was the only culprit, the rate of AL would be zero in all ICG cohorts. This emphasizes the multifactorial cause of AL. Nevertheless, real-time perfusion studies using ICG can be seen as an additional means of increasing patient's safety in colorectal surgery.
